# Prediabetes as a risk factor for major adverse cardiovascular events

**DOI:** 10.1080/07853890.2021.2000633

**Published:** 2021-11-11

**Authors:** Ramy Mando, Muhammad Waheed, Adrian Michel, Patrick Karabon, Alexandra Halalau

**Affiliations:** a Department of Cardiovascular Medicine, Beaumont Hospital Royal Oak, Royal Oak, MI, USA; b Oakland University William Beaumont School of Medicine, Rochester, MI, USA; c Department of Internal Medicine, Beaumont Hospital Royal Oak, Royal Oak, MI, USA; d Office of Research, Oakland University William Beaumont School of Medicine, Rochester, MI, USA

**Keywords:** Prediabetes, major cardiovascular events, haemoglobin A1c

## Abstract

**Introduction:**

Type II diabetes mellitus (DM) is a proinflammatory process and a known risk factor for major adverse cardiac events (MACE). The same inflammatory markers may be present in prediabetes (pDM); however, the relationship between pDM by HbA1c and MACE is not well studied. We sought to see if pDM increases one’s risk for MACE.

**Methods:**

We retrospectively studied patients at Beaumont Health, Michigan between 2006 and 2020. We divided patients into groups (G1–G5) based on haemoglobin A1c (HbA1c) trends over the study period as follows: G1: pDM patients who remained pDM; G2: pDM who progressed into DM; G3: pDM who normalized their HbA1c; G4: patients who maintained a normal HbA1c; and G5: patients with HbA1c persistently in the DM range. We compared MACE between the groups by univariate and multivariate regression analyses.

**Results:**

A total of 119,271 patients were included in the study (G1: *N* = 13,520, G2: *N* = 6314, G3: *N* = 1585, G4: *N* = 15,018, G5: *N* = 82,834). Pairwise comparison revealed a statistically significant increase in the odds of MACE in all groups compared to those with normal HbA1c values (G4; *p* < .001). After adjusting for baseline characteristics, multivariate regression revealed elevated odds of MACE in patients with persistent pDM (G1; aOR = 1.087, *p* = .002) and diabetes (G2/G5; aOR = 1.25 and aOR = 1.18, *p* < .001) compared to individuals with normal HbA1c values.

**Conclusion:**

Prediabetes is a risk factor for MACE. Normalization of HbA1c values appears to decrease the adjusted risk for MACE and should be the goal in patients with pDM.

**KEY MESSAGES**
- Patients with prediabetes (pDM) are at increased risk for major cardiovascular events.
- Normalization of HbA1c in pDM patients may have a clinically significant benefit, in terms of lowering the MACE risk.
- Prediabetes patients who progress into diabetes mellitus may represent a particularly high-risk group.

## Introduction

The World Health Organization estimated that in 2016 approximately 17.9 million deaths around the globe were due to cardiovascular disease (CVD), which continues to be a leading cause of death throughout the world [1]. Major adverse cardiovascular events (MACE) including cerebrovascular accidents (CVA), myocardial infarctions (MIs), unstable angina and acute heart failure are among the leading causes of mortality in the USA. According to the American Heart Association (AHA), it is estimated that about 655,000 people die each year in the United States because of CVD [2].

Type II diabetes mellitus (DM) is a known major risk factor associated with the development of CVD [3,4]. Individuals with DM are exposed to hyperglycaemia and many also have hyperinsulinaemia which has been associated with increased inflammatory and immunologic processes promoting deleterious vascular remodelling [5]. This increases the risk for cardiovascular complications like acute coronary syndrome (ACS) and CVA [5,6]. Prediabetes (pDM), or more recently termed, as “at-risk for diabetes”, identifies individuals that are at an intermediate stage between normal blood glucose tolerance and Type II DM [7]. The American Diabetes Association reports that as many as 70% of patients with pDM will progress to developing DM [8].

Several studies have established that inflammatory and immunologic processes that play a role in CVD in patients with DM are also found in those who have pDM. A study by Buysschaert et al. revealed that hyperglycaemia was associated with increased reactive oxidative species (ROS) [9–11]. These ROS in turn contribute to the pathogenesis of CVD seen in patients with impaired glucose metabolism [5]. Despite similar inflammatory states observed in patients with elevated HbA1c, cardiovascular risk calculators such as the Systemic Coronary Risk Estimation (SCORE) or race and sex-specific pooled cohort equation to estimate 10 year atherosclerotic cardiovascular disease (ASCVD) only include diabetes as a risk factor and fail to capture the risk conferred by HbA1c in pDM ranges [3,4,12].

We sought to examine patient level data to assess the risk for MACE in patients with pDM and compare incidence of events to those with persistent euglycaemia and diabetes. We also wanted to assess if there was a role for more aggressive management of pDM in the clinical setting based on incidence of MACE events in those with normalization of their prediabetic range haemoglobin A1c (HbA1c).

## Methods

### Study design and setting

This was an observational retrospective study conducted at Beaumont Health System, the largest healthcare systems in Southeast Michigan. Eight hospitals in Beaumont Health’s electronic health record (Epic System, Verona, WI) were queried between 1 January 2006 and 1 January 2020 to identify the study population. Data from the emergency room, inpatient and outpatient setting were included in our query. The Beaumont Health Institutional Review Board, # 2019-281, approved the current study.

### Participants

Patients were eligible to be included in the study if they were adults 18 years of age or older and had a minimum of two documented HbA1c results between 1 January 2006 and 1 January 2020. A normal HbA1c was defined as <5.7%. Prediabetes was defined as a HbA1c ≥5.7% and ≤6.4% [12]. Diabetes was defined as a HbA1c ≥6.5% [13,14]. Patients were subsequently divided into five groups (G1–G5) based on the trend between their first HbA1c and peak HbA1c as follows: group 1 (G1): patients with an initial HbA1c in the pDM range with all subsequent HbA1c maintained in the pDM range. Group 2 (G2): patients with an initial HbA1c in the pDM range with subsequent HbA1c values in the DM range. Group 3 (G3): patients with an initial HbA1c in the pDM range with subsequent HbA1c values that normalized. Group 4 (G4): normal control: patients with a HbA1c in the normal range throughout the study. Group 5 (G5) DM control: patients with a HbA1c in the DM range that remained in the DM range.

### Variables

The primary outcome of this study consisted of the composite major adverse cardiac events (MACE) defined as the composite of: ACS including non-ST and ST-elevation MI and unstable angina, MI, congestive heart failure (CHF), ischaemic stroke, percutaneous coronary intervention (PCI) and coronary artery bypass graft (CABG). We also assessed rates of all-cause mortality between groups. The data were queried using the International Classification of Diseases, Ninth and Tenth Revisions, Clinical Modification (ICD-9-CM and ICD-10-CM) codes and/or billing codes at the time of a hospital discharge.

The other variables of interest assessed included: patient age, gender, body mass index (BMI), baseline HbA1c, tobacco dependence, alcohol dependence, hypertension, hyperlipidaemia, peripheral vascular disease, anaemia, cancer, chronic kidney disease (CKD), chronic obstructive pulmonary disease (COPD), obstructive sleep apnoea (OSA), family history of MI, coronary artery disease, DM and stroke.

### Data sources/measurement and statistical analysis

The data including study population, variables of interest and outcomes, were extracted from the electronic health record with specific queries of patient problem lists, medical and surgical history, laboratory results, procedure notes, hospital primary diagnosis and hospital discharge diagnosis.

Continuous variables were presented as mean with standard deviation in parentheses and compared using the Student *t* test or Mann–Whitney’s *U* test (depending on normality). Categorical variables are expressed as frequencies with percentages in parentheses and compared using Chi-Square test. Pairwise comparisons between rates of MACE for each group were done using Chi-Square test. A stepwise multivariate logistic regression analysis was performed to determine whether classification into an individual’s respective group was associated with MACE. All-cause mortality was adjusted for age, gender and co-morbidities. The cumulative incidence rates of outcomes (including stroke, MI, CHF, ACS, CABG, PCI, cumulative MACE and mortality) across the five groups were estimated using Kaplan–Meier’s cumulative incidence curves and compared using log‐rank tests. Cox proportional hazards models were performed to estimate associations between MACE and HbA1c group during the study follow up, and hazard ratios (HRs) with corresponding 95% CIs were calculated for groups 1–5 with G4 serving as reference. A *p* value of  < .05 was considered statistically significant. All statistical analyses were conducted using the SPSS version 27.0 (SPSS Inc., Chicago, IL).

### Bias

In an effort to minimize selection bias, all patients identified in Beaumont’s healthcare database who met the inclusion criteria were included in the study. Information (measurement) bias was addressed by having all automated reports of patient data and outcomes generated by an individual who was not involved in the study protocol or statistical analysis. Data collection including variables of interest, outcomes and covariates were obtained in a standardized fashion without knowledge of patient groups. Moreover, to minimize inter-observer variability, regular meetings were held with our data collectors and biostatisticians to ensure all variables were obtained in a consistent and streamlined fashion. Researcher bias was controlled for by strict adherence to the study protocol. Nonetheless, the potential for unknown or unidentified confounders still remains.

## Results

### Patient demographics and comorbidities

**Figure 1.**
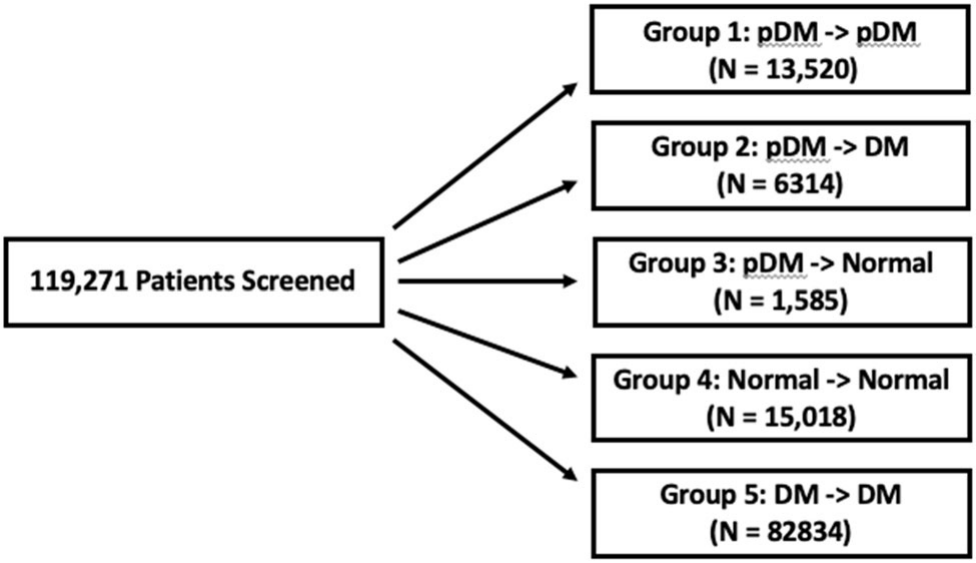
A total of 119,271 patients were included in the final analysis and categorized into their respective group based on their initial and peak HbA1c through the study.

A total of 119,271 patients were identified based on the inclusion criteria. Each patient was then categorized into one of five groups based on their HbA1c (Figure 1).

The average age for the study population was 62.4 years (SD = 14.4) and 48% (*N* = 56,983) of the population were male. The median follow-up time for the study population was 6.33 years (IQR 3.67, 8.24). There was a median of 7 HbA1c values per patient (IQR 4, 13). Baseline characteristics and comorbidities for the entire study population are summarized in Table 1. The same variables are listed in Table 2 categorized by group with the normal control serving as the reference.

**Table 1.** Characteristics of the study population.

Demographics	
Age (*N* = 119,271)	63.2 (IQR 53.2–72.5)
BMI (*N* = 119,271)	31.2 (IQR 27.1–36.5)
Male gender (*N* = 119,271)	56,983 (47.7%)
Race (*N* = 116,154)	White − 78,327 (67.4%)
	Black − 23,750 (20.4%)
	Other − 14,077 (12.2%)
Comorbidities	
Hypertension	75,464 (72.0%)
Dyslipidaemia	41,242 (39.4%)
Atrial fibrillation	7888 (7.5%)
COPD	9429 (9.0%)
CKD	8594 (8.2%)
Smoking history	22,085 (18.5%)
Alcohol history	53,054 (44.5%)
Cancer	9716 (9.3%)
Outcomes	
Death	3441 (3%)
MACE	43,403 (36.4%)
MACE count	0 − 75,868 (63.6%)
	1 − 8447 (7.1%)
	2 − 18,984 (15.9%)
	3 − 9596 (8.0%)
	4 − 5108 (4.3%)
	5 − 1156 (1.0%)
	6 − 112 (0.1%)
	Total: 102,087 events

BMI: body mass index; COPD: chronic obstructive pulmonary disease; CKD: chronic kidney disease; MACE: major adverse cardiovascular events.

**Table 2.** Characteristics of the study population by group.

Demographics						
Variable (% valid data)	Group 1 (pDM–pDM) (*N* = 13,520)	Group 2 (pDM–DM) (*N* = 6314)	Group 3 (pDM–N) (*N* = 1585)	Group 4 (N–N) (*N* = 15,018)	Group 5 (DM–DM) (*N* = 82,834)	*p* Value
Age (99.2%)	67.2 ± 12.7	66.4 ± 12.9	65.5 ± 14.8	58.0 ± 15.0	62.5 ± 14.4	<.01
BMI (96.3%)	31.3 ± 7.0	32.9 ± 7.1	30.9 ± 7.6	28.6 ± 6.7	33.2 ± 7.6	<.01
Male gender (99.2%)	5440 (40.2%)	3051 (48.3%)	608 (38.4%)	9174 (38.9%)	42,040 (50.8%)	<.01
Race (96.6%)						
Caucasian	9573 (71.6%)	4489 (71.7%)	1188 (75.6%)	12,014 (81.9%)	51,063 (63.6%)	
African American	2273 (17.0%)	981 (15.7%)	242 (15.4%)	1158 (7.9%)	19,096 (23.8%)	<.01
Other	1515 (11.3%)	792 (12.6%)	142 (9.0%)	1493 (10.2%)	10,135 (12.6%)	
Comorbidities						
Hypertension (87.1%)	8764 (70.1%)	4749 (79.6%)	907 (62.5%)	5600 (44.2%)	55,444 (76.8%)	<.01
Hyperlipidaemia (87.1%)	5966 (47.7%)	3062 (51.3%)	527 (36.3%)	3338 (26.3%)	28,349 (39.3%)	<.01
Atrial fibrillation (87.1%)	1120 (9.0%)	648 (10.9%)	135 (9.3%)	748 (5.9%)	5237 (7.3%)	<.01
COPD (87.1%)	973 (7.8%)	575 (9.6%)	120 (8.3%)	499 (3.9%)	7262 (10.1%)	<.01
CKD (87.1%)	769 (6.2%)	463 (7.8%)	118 (8.1%)	439 (3.5%)	6805 (9.4%)	<.01
Smoking history (99.2%)	2169 (16.0%)	1153 (18.3%)	261 (16.5%)	2004 (13.3%)	16,498 (19.9%)	<.01
Alcohol history (99.2%)	7702 (57.0%)	3396 (53.8%)	934 (58.9%)	9200 (61.3%)	31,822 (38.4%)	<.01
Cancer (87.1%)	1335 (10.7%)	665 (11.1%)	142 (9.8%)	989 (7.8%)	6585 (9.1%)	<.01
Outcomes						
Death (99.2%)	288 (2.1%)	178 (2.8%)	37 (2.3%)	170 (1.1%)	2768 (3.5%)	<.01
MACE (99.2%)	5286 (39.1%)	2797 (44.3%)	596 (37.6%)	3750 (25.0%)	30,974 (37.4%)	<.01

Total MACE events	12,340	6667	1392	8325	73,363
No events	8232 (60.9%)	3517 (55.7%)	989 (62.4%)	11,268 (75.0%)	51,860 (62.6%)
1 event	955 (7.1%)	486 (7.7%)	128 (8.1%)	808 (5.4%)	6070 (7.3%)
2 events	2444 (18.7%)	1248 (19.8%)	238 (15.0%)	1770 (11.8%)	13,284 (16%)
3 events	1195 (8.8%)	657 (10.4%)	149 (9.4%)	776 (5.2%)	6819 (8.2%)
4 events	562 (4.2%)	322 (5.1%)	65 (4.1%)	333 (2.2%)	3826 (4.6%)
5 events	116 (0.9%)	78 (1.2%)	15 (1.0%)	61 (0.4%)	886 (1.1%)
6 events	14 (0.1%)	6 (0.1%)	1 (0.06%)	2 (0.01%)	89 (0.11%)

BMI: body mass index; COPD: chronic obstructive pulmonary disease; CKD: chronic kidney disease; MACE: major adverse cardiovascular events.

### The impact of HbA1c on MACE and mortality

There were 102,087 MACE in 43,303 (36.3%) patients. This included 6475 (5.5%) ischaemic strokes, 33,893 (28.4%) MIs, 40,104 (33.6%) heart failure related admissions, 13,720 (11.5%) ACS, 2662 (2.2%) CABG and 5233 (4.8%) PCIs. MACE outcomes by group are summarized in Table 3.

**Table 3.** MACE and all-cause mortality by HbA1c defined groups.

	Group 1 (pDM–pDM) (*N* = 13,520)	Group 2 (pDM–DM) (*N* = 6314)	Group 3 (pDM–N) (*N* = 1585)	Group 4 (N–N) (*N* = 15,018)	Group 5 (DM–DM) (*N* = 82,834)	*p* Value
MACE (% within group)	5286 (39.1%)*	2792 (44.3%)*	596 (37.6%)*	3750 (25%)	30,974 (37.4%)*	*p* < .001
	1.392 [1.355–1.431]*	1.331 [1.301–1.361]*	1.065 [1.052–1.079]*		1.79 [1.725–1.867]*	
Stroke	866 (6.4%)*	428 (6.8%)*	109 (6.9%)*	601 (4.0%)	4471 (5.4%)*	*p* < .001
	1.30 [1.221–1.384]*	1.216 [1.154–1.281]*	1.072 [1.038–1.106]*		1.369 [1.225–1.493]*	
Myocardial infarction	4224 (31.2%)*	2246 (35.6%)*	458 (28.9%)*	2873 (19.1%)	24,092 (29.1%)*	*p* < .001
	1.399 [1.357–1.443]*	1.335 [1.301–1.370]*	1.061 [1.046–1.076]*		1.734 [1.660–1.811]*	
Heart failure	4820 (35.7%)*	2595 (41%)*	538 (33.9%)*	3354 (22%)	28,797 (34.8%)*	*p* < .001
	1.396 [1.357–1.436]*	1.345 [1.313–1.378]*	1.065 [1.050–1.079]*		1.853 [1.779–1.931]*	
ACS	1686 (12.5%)*	935 (14.8%)*	199 (12.6%)*	1053 (7.0)	9847 (11.9%)*	*p* < .001
	1.408 [1.341–1.478]*	1.363 [1.306–1.422]*	1.082 [1.055–1.109]		1.789 [1.675–1.911]*	
PCI	490 (3.6%)*	284 (4.5%)*	55 (3.5%)*	285 (1.9%)	4119 (5.0%)*	*p* < .001
	1.443 [1.315–1.584]*	1.417 [1.305–1.538]*	1.081 [1.031–1.133]		2.705 [2.396–3.054]*	
CABG	254 (1.9%)*	179 (2.8%)*	33 (2.1%)*	159 (1.1%)	2037 (2.5%)*	*p* < .001
	1.372 [1.214–1.551]*	1.505 [1.343–1.685]*	1.093 [1.025–1.166]		2.356 [2.003–2.771]*	
Total MACE	12,340*	6667*	1392*	8325	73,363*	*p* < .001
MACE events/patient	0.91*	1.06*	0.88*	0.55	0.89*	*p* < .001
All-cause mortality (%)	288 (2.1%)*	178 (2.8%)*	37 (2.3%)*	170 (1.1%)	2768 (3.5%)*	*p* < .001
	1.422 [1.262–1.603]*	1.447 [1.299–1.621]*	1.102 [1.034–1.175]*		3.174 [2.716–3.709]*	

MACE: major adverse cardiovascular events; ACS: acute coronary syndrome; PCI: percutaneous coronary intervention; CABG: coronary artery bypass surgery.

* *p* < .01 compared to group 4 (normal control); OR with 95% confidence intervals reflective of comparison to the normal control group.

**Figure 2.**
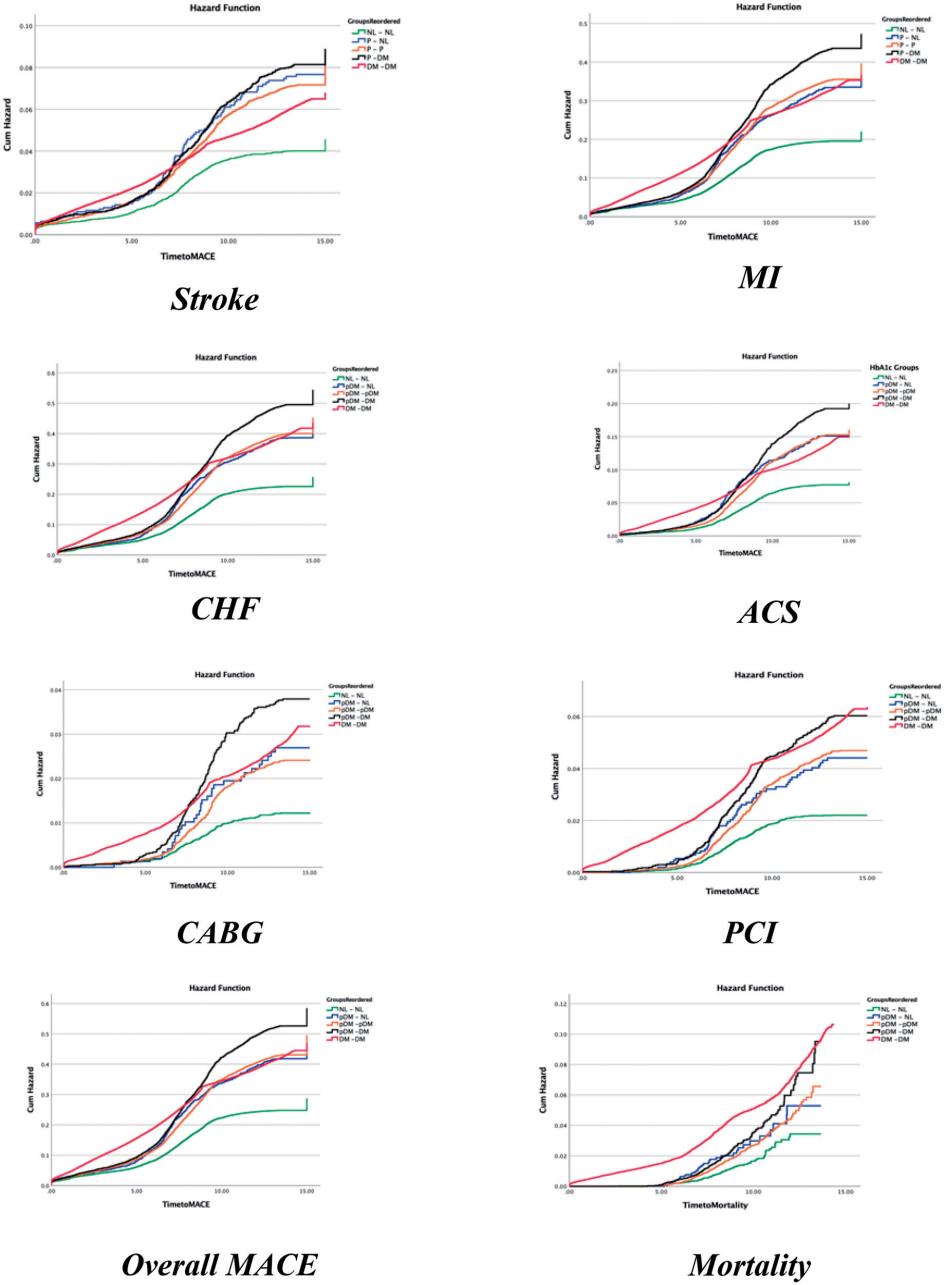
Kaplan–Meier’s survival curves of MACE and mortality according to HbA1c group. MI: myocardial infarction; CHF: congestive heart failure; ACS: acute coronary syndrome; CABG: coronary artery bypass graft; PCI: percutaneous coronary intervention.

Unadjusted analysis showed that when compared to normal (G4), patients with HbA1c persistently in the pDM range (G1) were at higher risk for MACE (OR 1.93, 95% CI [1.834–2.029], *p* < .001). This trend was also appreciated when comparing G2, G3 and G5 (*p* < .001) to G4, normal HgA1c patients. These differences are illustrated by Kaplan–Meier’s plots comparing incidence of stroke, MI, CHF, ACS, CABG, PCI, overall MACE and mortality between the groups (*p* < .001; Figure 2). Overall, compared to patients with normal A1c, prediabetic and diabetics had elevated risk for MACE and mortality.

**Figure 3.**
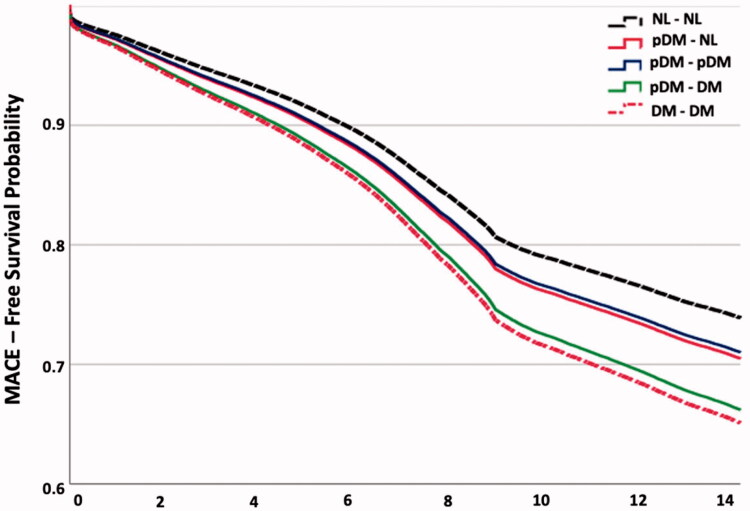
Adjusted cumulative 15 years incidence of MACE.

After adjusting for age, BMI, gender, hypertension, hyperlipidaemia, atrial fibrillation, peripheral artery disease, CKD, COPD, smoking status and alcohol use in the logistic regression model, we have found an increased odds of MACE in G1 (OR 1.09, 95% CI [1.02–1.15], *p* = .011) and G5 (OR 1.18, 95% CI [1.027–1.15], *p* = .03). Patients who were initially pDM with normalization of HbA1c (G3) had no significant difference in adjusted odds for MACE compared to normal (G4) (*p* = .393) (Table 4). Hazard ratios were analysed and revealed a statistically significant increase in risk of MACE between the groups compared to G4 (Table 4, Figure 3).

**Table 4.** Associations of MACE using bivariate logistic regression analysis and Cox regression patients with normal HbA1c as reference (G4).

	Odds ratio [95% CI]	*p* Value
Age	0.95 [0.92–0.96]	<.01
BMI	1.17 [0.85–1.27]	.88
Male gender	1.35 [1.30–1.40]	<.01
Hypertension	1.83 [1.76–1.91]	<.01
Hyperlipidaemia	1.03 [0.94–1.12]	.132
Atrial fibrillation	2.63 [2.44–2.83]	<.01
Peripheral artery disease	1.82 [1.36–2.44]	<.01
Chronic kidney disease	2.02 [1.90–2.15]	<.01
COPD	2.01 [1.90–2.15]	<.01
Smoking status	1.98 [1.67–2.31]	<.01
Alcohol use	1.37 [1.24–1.43]	.04

	Unadjusted odd ratio	*p* Value	Adjusted odd ratio	*p* Value	Adjusted hazard ratio	*p* Value
Group 1 (*N* = 13,520)	1.929 (1.834–2.029)	*p* < .001	1.087 (1.02–1.15)	*p* = .011	1.13 (1.09–1.18)	*p* < .01

Group 2 (*N* = 6314)	2.390 (2.246–2.542)	*p* < .001	1.25 (1.14–1.36)	*p* = .015	1.36 (1.30–1.43)	*p* < .01

Group 3 (*N* = 1585)	1.811 (1.625–2.018)	*p* < .001	0.974 (0.856–1.109)	*p* = .393	1.16 (1.03–1.26)	*p* = .043

Group 5 (*N* = 82,834)	1.80 (1.725–1.867)	*p* < .001	1.18 (1.087–1.232)	*p* = .03	1.419 (1.37–1.47)	*p* < .01


## Discussion

The main findings of this study suggest that compared to patients with normal HbA1c: (1) patients with pDM are at increased risk for MACE; (2) patients whose HbA1c decreased from the pDM range to normal range experienced fewer cardiovascular events compared to patients with persistent HbA1c in the pDM range; (3) pDM who progress into DM may represent a particularly high-risk group; (4) patients with DM have significantly higher MACE rate and mortality.

Type II DM is a well-known risk factor for cardiac events. Tenerz et al. investigated the prevalence of DM among patients with acute MIs and found that one out of four patients with acute MIs had DM [15]. Additionally, the BARDOT trial examined CAD in asymptomatic diabetics and found that about 22% of diabetic patients, all of whom had no history or symptoms of CAD, had an abnormal baseline myocardial perfusion single-photon emission computed tomography. Furthermore, the study found these patients also had a sevenfold higher rate of progression to “overt or silent CAD”, despite medical therapy. However, the impact of pDM has not been strongly established [16].

Our findings are consistent with recent literature in regard to the potential clinical association between pDM and CVD. It has been shown that cardiovascular and renal diseases are more prevalent in the pDM population, and that this population of patients may represent an opportunity to reduce cardiovascular related morbidity [17]. A meta-analysis of 53 prospective cohort studies found that when compared to normoglycaemia, pDM was associated with an increased risk of composite CVD, coronary heart disease, stroke and all-cause mortality [18]. A more recent meta-analysis by Cai et al. of 129 studies found that pDM was associated with nearly a 15% increased risk of CVD and 13% increased risk of all-cause mortality when compared to individuals with normoglycaemia [19]. Expanding upon their prior research, Cai et al. recently published a large meta-analysis including almost 10 million individuals with a median follow up time of 8 years revealing that, compared to normoglycaemia, patients with pDM had as high as a 58% increased risk for heart failure [20]. Moreover, Mai et al. looked at 12 studies including 28,643 patients with heart failure and noted significantly increased risk for all-cause mortality, cardiovascular mortality, heart failure hospitalization in patients with pDM [21]. Rossello et al. recently published a study highlighting the increased incidence of subclinical atherosclerosis diagnosed by two-dimensional vascular ultrasound and noncontrast cardiac computed tomography after adjusting for established cardiovascular risk factors in patients with abnormal HbA1c values [22]. This study reaffirmed the previous finding by Scicali et al. showing elevated HbA1c in non-diabetic patients was associated with increased coronary calcium scores and peripheral atherosclerotic burden [23]. Epidemiological studies such as the DECODE and Funagata Diabetes studies have also pointed towards the association between pDM and CVD [24–26]. The results and outcomes described in these studies strongly support our findings and point to pDM as an independent risk factor for adverse cardiac events.

In the present study, we observed a consistent and reproducible increase in the incidence of CVA, MI, CHF admission, ACS, CABG and PCI in G1 and G5 as illustrated by the Kaplan–Meier plots (*p* < .01). Furthermore, pDM remained a significant risk factor for MACE after adjusting for age, gender, smoking status, hypertension, hyperlipidaemia, peripheral artery disease and CKD. These findings suggest that even mild to moderate elevations in blood glucose may trigger the same microvascular changes seen in DM. Normalization of HbA1c (G3) was found to be associated with reduction in the odds of developing MACE to a level similar to those in the normal control (G4; OR 0.974, *p*=.39).

**Figure 4.**
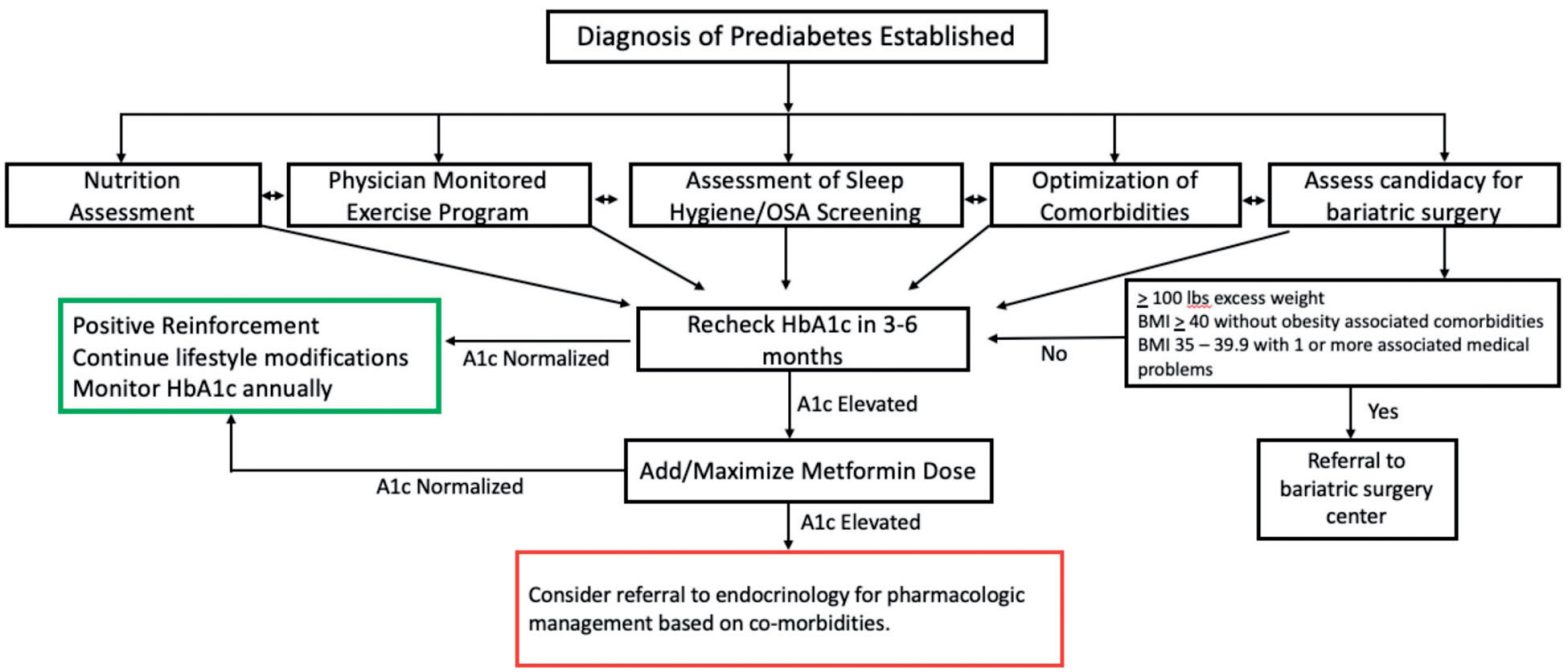
Proposed treatment algorithm for patients with prediabetes.

The results of our study offer insight to an area not well explored and may have significant clinical implications. We feel that our results should increase physician awareness to the pDM condition and its potential long-term complications. Based on the results of this study, we would favour aggressive treatment of pDM. This would include behavioural modification, nutritional assessment/intervention, pharmacologic therapy, optimization of risk factors including, but not limited to, hypertension, hyperlipidaemia, OSA, respiratory conditions and orthopaedic issues (i.e. gout, osteoarthritis, etc.) which may limit one’s ability to exercise (Figure 4).

There are currently no established guidelines for the primary or secondary prevention of CVD in patients with pDM. Furthermore, current CVD risk assessment tools and algorithms, including the ASCVD and SCORE risk estimators, recommended by the European Society of Cardiology and U.S. guidelines do not account for pDM in their CVD risk assessments [3,4,12]. The American Diabetes Association has mentioned that pDM should not be viewed as a clinical entity which often leaves patients undiagnosed, unaware and without intervention to prevent diabetes or CVD [13]. We feel that our study, as well as several others, some of which previously discussed, suggest that pDM is in fact its own clinical entity with associated morbidity that is often left undertreated. Well-designed prospective studies with longer-term follow-up are needed to best assess the implications of pDM on MACE and mortality.

## Limitations

This study has several limitations. As a retrospective observational analysis, this study evaluated association and not causation. Other study designs such as randomized controlled trials are needed to validate the association between pDM and major adverse cardiovascular events. The potential for selection bias should also be considered as the data were collected from one health system electronic medical record (EMR). It was not possible to adjust for all confounding variables in our retrospective analysis and it is possible that there may have been unmeasured underlying risk factors that could have had an impact on our study outcomes. Inclusion criteria did not require restriction of the baseline CVD contributing to increased clinical heterogeneity within the study population. Due to the large study population, we were not able to collect data regarding whether individuals classified within the study as “normal” or “pDM” may have met criteria for DM by other criteria (i.e. glucose tolerance testing) or during gestation. Also, the authors were not able to individually review each chart to assess for haemolysis or transfusions at the time of the HbA1c results, or for falsely high or low HbA1c in haemoglobinopathies, anaemia, bariatric surgery, that could lead to overtreatment or undertreatment of diabetes. For similar reasons, individual medical therapy regimens were not collected as it was felt that the temporal association of starting medications would be difficult to account for and not feasible.

## Conclusions

In this study, we found that individuals who have a HbA1c outside the normal range (pDM and DM) are at increased risk for MACE and mortality. Physicians should be aware and advocate for a more aggressive treatment strategy in pDM patients, with the aim of normalizing the HgA1c as soon as possible.

## Ethical approval

## Data Availability

Raw data were generated at Beaumont Health. Derived data supporting the findings of this study are available from the corresponding author [AH] on request.
